# Monoclonal Antibodies Against Vascular Endothelial Growth Factor A (VEGF-A) Reduce Synovitis, Bone Damage, and Osteogenesis in an SKG Mouse Model of Spondyloarthritis

**DOI:** 10.1155/jimr/8870895

**Published:** 2025-05-30

**Authors:** Marcin Czepiel, Małgorzata Stec, Anna Gąsiorek, Anna Gałuszka, Kornelia Kłosińska, Joanna Kozieł, Jarosław Czyż, Jarosław Baran, Przemysław Błyszczuk, Maciej Siedlar, Mariusz Korkosz

**Affiliations:** ^1^Department of Clinical Immunology, Institute of Pediatrics, Jagiellonian University Medical College, Wielicka 265 Str., Krakow, Poland; ^2^Department of Microbiology, Faculty of Biochemistry, Biophysics and Biotechnology, Jagiellonian University, Gronostajowa 7 Str., Krakow, Poland; ^3^Department of Basic Sciences, Faculty of Veterinary Medicine, University of Agriculture in Krakow, Rędzina 1c Str., Krakow, Poland; ^4^Department of Cell Biology, Faculty of Biochemistry, Biophysics and Biotechnology, Jagiellonian University, Gronostajowa 7 Str., Krakow, Poland; ^5^Department of Rheumatology and Immunology, Jagiellonian University Medical College, Jakubowskiego 2 Str., Krakow, Poland

## Abstract

Vascular endothelial growth factor-A (VEGF-A) plays a pivotal role in inflammatory rheumatic diseases, including spondyloarthritis (SpA). Recently, we have demonstrated that the expression of VEGF-A in human classical monocytes is positively associated with the number of swollen and painful joints in SpA patients. Therefore, we tested whether the anti-VEGF-A therapy can affect the hallmarks of SpA in the SKG mouse model. When initiated at the disease onset, the administration of anti-VEGF-A monoclonal antibodies (mAbs) significantly reduced the objective symptoms of SpA in the curdlan suspension-treated mice compared to their untreated and isotypic control-treated counterparts. Micro-computed tomography (CT) imaging revealed substantial benefits of the treatment, with anti-VEGF-A mAbs-treated mice exhibiting preserved joint spaces, reduced number and depth of bone erosions, and limited new bone formation in hind paws, calcaneus, sacroiliac joints, and caudal vertebrae. These effects remained in contrast to the pronounced damage and osteogenesis in relevant skeletal regions of control animals. The histological assessment confirmed reduced synovial inflammation and bone erosions in anti-VEGF-A mAbs-treated mice, underscoring the efficacy of the treatment in mitigating SpA bone damage. Collectively, anti-VEGF-A mAbs treatment favors the maintenance of joint and spine structures, alleviates bone destruction and osteogenesis, and reduces local inflammation in the mouse SpA model. Our study pinpoints anti-VEGF-A mAb therapy as a promising avenue to understand the SpA pathogenesis and as a treatment option. It also addresses vascular and inflammatory aspects of the disease and illustrates the potential of the SKG mouse SpA model for assessing the long-term safety of anti-VEGF-A therapy before its clinical translation.

## 1. Introduction

Vascular endothelial growth factor A (VEGF-A) is a multifaceted signaling protein known for its pivotal role in angiogenesis; however, its functions reach beyond vascular growth. VEGF-A, a member of the VEGF family, exerts pleiotropic effects in various physiological and pathological processes [1]. In addition to promoting endothelial cell proliferation, migration, and survival, crucial for vasculature development and tissue repair, VEGF-A plays a role in neuroprotection, influencing neuronal survival and regeneration [2, 3]. Moreover, VEGF-A is involved in immune modulation, affecting immune cell recruitment and activity at sites of inflammation, as well as contributing to tissue homeostasis and repair [4, 5]. Dysregulation of VEGF-A has been linked to several diseases, including cancer, retinopathies, and several inflammatory rheumatic disorders [1, 6].

Spondyloarthritis (SpA) constitute a group of rheumatologic disorders characterized by persistent inflammation in the spinal column, peripheral joints, and entheses, leading to adverse skeletal remodeling [7]. The axial subform of SpA (axSpA), primarily affecting the sacroiliac joints and spine, is distinguished by an excessive formation of new bone, resulting in significant biomechanical impairment and disability [8]. On the other hand, the peripheral subtype (perSpA) is predominantly characterized by synovitis in peripheral joints, enthesitis, and dactylitis. In SpA, VEGF-A has been found to be upregulated in the synovial tissue and fluid, as well as in the serum of patients [9–11]. The exact mechanisms by which VEGF-A contributes to the pathogenesis of SpA are not fully understood. However, several lines of evidence suggest that VEGF-A may induce the expression of adhesion molecules in the synovial microenvironment and promote the recruitment of inflammatory cells into joint soft tissue [12, 13] or stimulate the differentiation of osteoclasts, leading to bone resorption, erosion, and joint damage [14, 15].

Given the role of VEGF-A in the pathogenesis of SpA, there has been interest in targeting this protein as a therapeutic approach [6]. More basic research is needed to determine the mode of action and efficacy of VEGF-A-targeted therapy in SpA. Here, we demonstrate the therapeutic effects of the application of monoclonal anti-VEGF-A antibodies in an SKG animal model of SpA. SKG mice harbor a specific mutation in the ZAP-70 gene (affecting T-cell receptor signaling) that makes them susceptible for developing SpA upon a single intraperitoneal injection of *β*-glucan [16, 17]. This animal model closely mimics the complexity of human SpA disease pathogenesis and its clinical features (including peripheral arthritis, spondylitis, dactylitis, enthesitis, and psoriatic skin lesions), making it especially suitable for studying disease pathomechanisms and designing therapies against SpA [16, 18].

## 2. Materials and Methods

### 2.1. Animals

SKG mice [16, 17] used in this study were kindly provided by dr Erik Lubberts (ErasmusMC, the Netherlands) after agreement with dr Ranjeny Thomas (The University of Queensland, Australia). The use of experimental animals in this research was carried out in accordance with relevant guidelines and regulations and approved by the local bioethics committee in Cracow (Poland)—approval number 710/2023. 10-week-old female SKG mice were used in the experiment. Throughout the experiment, mice were housed with a 12-h light/12-h dark cycle and fed a normal mouse diet ad libitum. For the experiments, mice were divided into four study groups: (a) no disease induction (*n* = 3), (b) disease induction, no treatment (*n* = 10), (c) disease induction, isotype antibody treatment (*n* = 10), and (d) disease induction, anti-VEGF-A antibody treatment (*n* = 10).

### 2.2. Disease Induction and Disease Severity Scoring

The SpA-like disease was induced in SKG mice by a single intraperitoneal injection of curdlan suspension (3 mg per mouse diluted in 200 µl of phosphate-buffered saline [PBS]). No anesthetics were employed in the study, as anesthesia procedures could have caused more distress to the animals than the experimental procedures themselves. The disease signs (initially redness and swelling of the digits) started to appear ~1 week postdisease induction. Treatment was either with anti-VEGF-A monoclonal antibodies (mAbs) against murine VEGF-A (clone: G6-31; isotype: Mouse IgG2a; Creative Biolabs) or control immunoglobulin of the same isotype (Mouse IgG2a Isotype control antibody; Creative Biolabs) (50 µg of antibody diluted in 200 µl of physiological salt) injected intraperitoneally on days 8, 11, 14, and 17 postdisease induction. The disease severity score (peripheral arthritis score) was assessed as follows: 0 = no swelling or redness, 0.1 = swelling or redness of the digits, 0.5 = mild swelling and/or redness of the wrist or ankle joints, and 1 = severe swelling of large joints. Maximal score = 6. Around day 70 of the experiment, the mice were euthanized by cervical dislocation by a trained professional and subjected to further analyses postmortem.

### 2.3. Micro-Computed Tomography (CT) Scanning

The limbs and spine (including sacroiliac joints) of euthanized animals were dissected and fixed with 4% paraformaldehyde (PFA) for 1 week and then stored in PBS for subsequent scanning. The surrounding connective tissues and muscles were removed before scanning. Specimens were then scanned with a high-resolution computed tomography scanner (MILabs, The Netherlands) at 20 µm resolution. A 3D reconstruction and analysis of the micro-CT images were performed with the 3D slicer software. Images were independently assessed by two specialists with expertise in SpA imaging blinded to the treatment (rheumatologist and radiologist). For analysis of trabecular bone parameters such as trabecular bone volume fraction (BV/TV), trabecular thickness (Tb.Th), trabecular number (Tb.N), connectivity (the number of connected structures, i.e., trabeculae in a trabecular network), connectivity density (number of trabeculae per unit volume) the BoneJ2 software package for ImageJ was used [19].

### 2.4. Histological Stainings and Assessment

#### 2.4.1. Hematoxylin and Eosin (H&E) Staining

The hind limbs and lumbar vertebrae of euthanized animals were dissected and fixed with 4% PFA for 1 week. Following 2 weeks of decalcification in 20% EDTA in 4% PFA, specimens were embedded in paraffin, sectioned into 10 μm slices, and stained with H&E solution.

Synovial inflammation and bone erosions in diseased joints were scored as follows:

a. For synovial inflammation: 0—healthy 1–2 cell layers of synovial membrane, no inflammatory infiltrates; 1—3–5 layers of synovial membranes, mild cellular infiltrates into synovium and exudate in the joint cavity; 2—multilayered synovial cell membranes, enhanced cellular infiltrates and increased density throughout the joints; 3—maximal expanded inflammation filling all joint cavities, hyperplastic synovial tissue, high cell density; maximal score = 3;
b. for bone erosions in diseased joints: 0—healthy, intact bone surface; 1—small, superficial bone erosion within outer surface of the cortical bone; 2—enhanced focal, subchondral bone erosions, partial or complete penetration through cortical bone, small breakthrough to bone marrow; 3—massive enlarged subchondral bone erosion, extended synovial pannus invasion mostly causing complete breakthrough of cortical bone to the bone marrow, loss of bone architecture; maximal score = 3.

#### 2.4.2. Safranin O/Fast Green Staining

Rehydrated tissue sections were immersed in Weigert's iron hematoxylin for 1 min, followed by rinsing in distilled water until the sections appeared clear. The sections were then sequentially stained with 0.02% Fast Green for 5 min, 1% acetic acid for 30 s, and 0.1% Safranin O for 10 min. After staining, they were dehydrated and mounted with a mounting medium.

Osteoarthritic cartilage damage in hind paws (a) and lumbar spine (b) was assessed according to Osteoarthritis Research Society International (OARSI) criteria [20, 21] as follows:

a. For paws [20]: 0—Normal, 0.5—Loss of Safranin-O without structural changes, 1—Small fibrillations without loss of cartilage, 2—Vertical clefts down to the layer immediately below the superficial layer and some loss of surface lamina, 3—Vertical clefts/erosion to the calcified cartilage extending to <25% of the articular surface, 4—Vertical clefts/erosion to the calcified cartilage extending to 25%–50% of the articular surface, 5—Vertical clefts/erosion to the calcified cartilage extending to 50%–75% of the articular surface, 6—Vertical clefts/erosion to the calcified cartilage extending >75% of the articular surface
b. For the lumbar spine following parameters of intervertebral disk regions have been assessed [21]: nucleus pulposus (NP, cellularity and morphology, fibrosis, matrix organization—max score = 3), annulus fibrosus (AF, cellularity, bulging, lamellar organization, clefts/fissures—max score = 3), endplate (cellularity, fissures/microfractures, presence of Schmorl's nodes—max score = 2), interface (cellularity, NP-AF boundary, NP-EP boundary, and the AF lamella disruption into the endplate—max score = 2). Finally, an average of all scores has been calculated.

### 2.5. Statistical Analysis

For peripheral arthritis score comparison, the two-way ANOVA was used with Bonferroni's multiple comparisons test (normally distributed data). For histology and uCT datasets, the nonparametric Kruskal–Wallis test was used for statistical analysis (not normally distributed data). Differences were considered statistically significant at *p* < 0.05. All analyses were performed with the Prism 6 software (GraphPad).

## 3. Results

### 3.1. Anti-VEGF-A Therapy Reduces the Severity of SpA-Like Disease in an SKG Mouse Model

**Figure 1:**
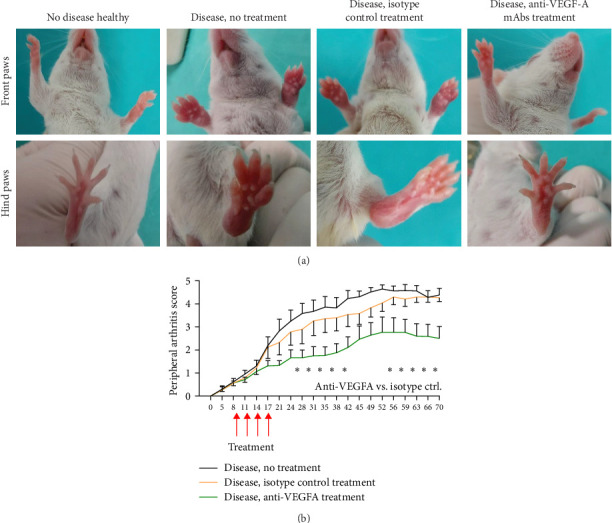
SpA-like disease progress in SKG mice. (A) Macroscopic images of paws of healthy and SpA-induced SKG mice with redness and swelling visible in diseased animals. (B) Disease progression (peripheral arthritis score) in relevant study groups (*n* = 10). Treatment dosing is indicated as red arrows. Error bars are the standard error of the mean. Asterisks represent the statistically significant difference between peripheral arthritis score for anti-VEGF-A mAb-treated and isotype-treated animals (two-way ANOVA with Bonferroni's multiple comparisons test; *⁣*^*∗*^*p* ≤ 0.05).

SpA therapy with anti-VEGF-A mAbs was initiated at the onset of disease signs in the second week after disease induction. Most of the animals already demonstrated some SpA signs, of which the redness and swelling of the digits were the most noticeable. As the disease progressed, the redness of the tails and ears, as well as swelling of the larger joints (i.e., ankles and wrists), became evident (Figure 1A). Most severely affected mice (peripheral arthritis score of 4 or higher) demonstrated substantial swelling of all four paws, though they did not need any assistance in reaching food or drinking water. Treatment of mice with four consecutive doses of anti-mouse VEGF-A mAbs resulted in significant long-term reduction of disease severity (as revealed by peripheral arthritis score) compared to untreated animals as well as mice treated with the same doses of nonspecific mAbs of the same isotype (Figure 1B).

### 3.2. Bone Destruction and New Bone Formation as Revealed by Micro-CT Imaging

**Figure 2:**
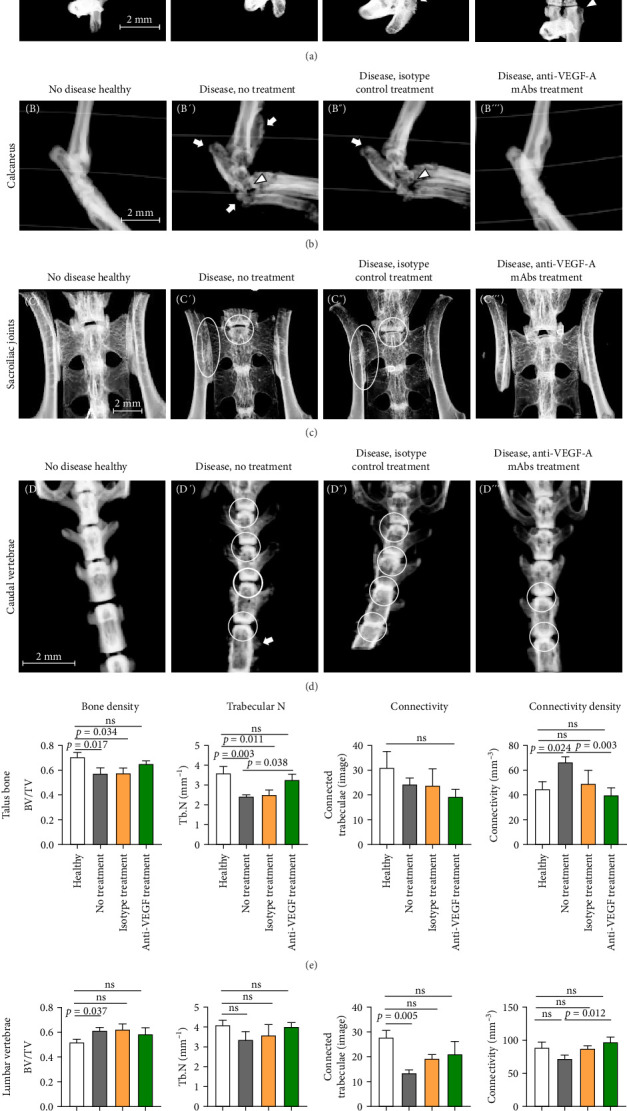
Micro-CT imaging of skeletal structures in SKG mice. Micro-CT images of hind paws, calcaneus, sacroiliac joints, and caudal vertebrae (A, B, C, and D, respectively) were taken postmortem. A detailed description of the observed changes is provided in the main text. Arrows indicate new bone formation; arrowheads indicate bone erosions; encircled areas indicate loss of joint/disc space. (E, F) Trabecular bone parameters of the talus (E) and lumbar vertebrae (F). BV/TV—trabecular bone volume fraction; Tb.N—trabecular number; connectivity—number of connected structures (i.e., trabeculae in the trabecular network); connectivity density—number of trabeculae per unit volume. Details are provided in the Materials and Methods section (mean with SEM; *n* = 6; Kruskal–Wallis test; ns, not significant). Scale bars apply to entire rows.

**Figure 3:**
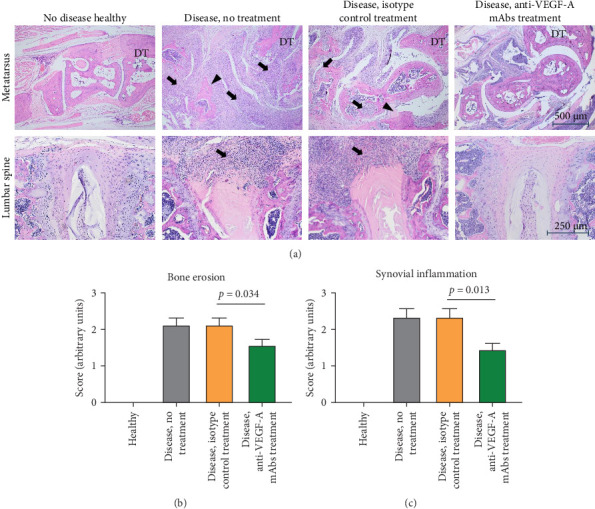
Histological assessment of synovial inflammation and bone erosion in SKG experimental groups. (A) H&E staining of metatarsus (upper panel) and lumbar spine (lower panel) tissue sections. Extensive inflammatory infiltrates visible within the ankle (upper panel) and intervertebral discs (lower panel) in mice from no treatment and isotype control treatment groups. Cellular infiltrates are indicated by arrows; synovial pannus formation is indicated by arrowheads. (B) and (C) Bone erosion and synovial inflammation scores assessed in the metatarsus of SKG mice based on H&E staining (mean with SEM; *n* = 9; Kruskal–Wallis test). DT, distal tibia. Scale bars apply to entire rows.

**Figure 4:**
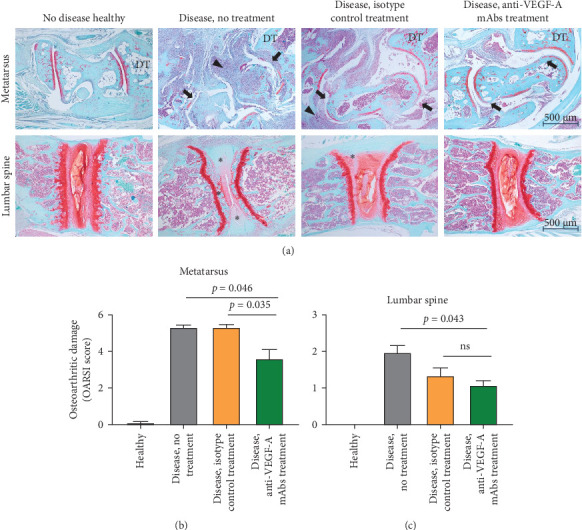
Histological assessment of osteoarthritic damage in SKG experimental groups. (A) Safranin O/Fast Green staining of metatarsus (upper panel) and intervertebral area of lumbar spine (lower panel) tissue sections. Cartilage is stained red/orange; bone is stained blue. Upper panel: extensive tissue damage with accompanied immune cell influx (arrowheads) apparent in nontreated and isotype control-treated animals. Thinning/loss of Safranin O staining (arrows) indicating cartilage erosion/damage appears extensive in no treatment and isotype control treatment groups and to a lesser extent for anti-VEGF-A treated animals. Lower panel: cartilage tissue ossification (asterisks) and degradation are clearly visible in the nontreatment group and, to a lesser extent, in the isotype control treatment group. (B) and (C) Quantification of osteoarthritic damage of metatarsus region (B; talus bone) and intervertebral area of lumbar spine (C) based on OARSI criteria (M&M section; mean with SEM; *n* = 9; Kruskal–Wallis test). DT, distal tibia. Scale bars apply to entire rows.

Micro-CT imaging of SKG mice revealed bone destruction and new bone formation in peripheral joints. Within hind paws, signs of bone destruction were observed in diseased, nontreated, and isotype-treated animals (Figure 2A',A”), for example, decreased trabecular bone density, narrowing/loss of joint space, erosions (tarsal bones, metatarsophalangeal, interphalangeal, proximal, and distal interphalangeal joints) and inflammatory subchondral cysts. New bone formation was evident at the proximal phalanxes (Figure 2A',A”). Conversely, in anti-VEGF-A mAb-treated mice, joint spaces were generally preserved, and only minor signs of bone erosion and new bone formation were detected, along with some periarticular trabecular bone loss (Figure 2A”'). Decreased bone density and erosions within calcaneus at the insertion area of the Achilles tendon were evident in diseased, nontreated, and isotype-treated animals (Figure 2B',B”). In contrast, animals treated with anti-VEGF-A mAbs maintained almost intact bone structure (Figure 2B”'). In diseased, nontreated, and isotype-treated animals, a substantial decrease of trabecular bone density and thinning of cortical bone accompanied by narrowing of joint spaces was detected in sacroiliac joints (Figure 2C',C”). In comparison, anti-VEGF-A mAb treatment resulted in the maintenance of the joint space and cortical thickness but with a slight loss of trabecular bone density (Figure 2C”'). Similarly, in caudal vertebrae, compared to healthy animals (Figure 2D), the diseased, nontreated, and isotype-treated mice demonstrated a substantial decrease in trabecular bone density, disc space narrowing, and some evidence of new bone formation on the vertebral bodies (Figure 2D',D”). On the contrary, in caudal vertebrae of anti-VEGF-A mAb-treated mice, preservation of disc space was observed, but a decrease in trabecular bone density was apparent (Figure 2D”'). Trabecular bone parameters such as bone volume fraction (BV/TV), trabecular number (Tb.N), connectivity connectivity density (Conn.D) of the talus bone showed no significant differences between healthy and anti-VEGF-A mAb-treated animals (Figure 2E). On the other hand, untreated as well as isotype-treated mice displayed significantly lowered BV/TV (*p*=0.017 and *p*=0.034, respectively) and Tb.N (*p*=0.003 and *p*=0.011, respectively) compared to healthy animals (Figure 2E) suggesting much more severe rearrangement of trabecular bone structure, which is consistent with higher degree of inflammation and cartilage damage observed within ankle joints of these animals (Figures 3 and 4, respectively). The differences were not as robust for lumbar vertebrae bodies, as only untreated mice displayed significant differences in BV/TV (*p*=0.037) and connectivity parameters (*p*=0.005 for connectivity and *p*=0.012 for connectivity density) compared to healthy animals (Figure 2F). Still, the Tb.N of these mice, although lowered, did not differ significantly between any of the studied groups (Figure 2F). The above results might suggest that the axial skeleton is not affected to the same extent by induced SpA-like conditions as a peripheral skeleton in the described SKG model.

### 3.3. Histological Assessment of Synovial Inflammation and Bone/Cartilage Erosion

Intraperitoneal administration of curdlan in SKG mice promotes the development of SpA features, including peripheral arthritis (synovitis, dactylitis), enthesitis, and spondylitis. After disease induction, an inflammatory infiltrate appeared in the synovium, entheses, ligaments, and intervertebral discs, along with bone erosions in peripheral joints. Thus, the extent of synovial inflammation and bone erosions were assessed within longitudinal sections of the feet (metatarsus) of the hind limbs, as well as cellular infiltrates within the outer layer of discs in the lumbar spine. In healthy animals (no disease induction), no signs of inflammatory infiltration were detected, along with intact cortical bone and intervertebral discs. On the contrary, in animals that did not receive treatment or received isotype control treatment, enhanced cellular infiltrates were revealed in the synovium and intervertebral discs and exudate in the joint cavity. In addition, joint tissue in these animals manifested with extended synovial pannus invasion, causing a breakthrough of cortical bone to the bone marrow cavity (erosions) and loss of bone architecture (Figure 3A). In anti-VEGF-A mAb-treated mice, synovial and intervertebral disc infiltration and bone structure damage were also evident, but to a substantially lesser extent (Figure 3A). In general, anti-VEGF-A mAb treatment significantly reduced synovial inflammation (*p*=0.013) and the extent of bone erosion (*p*=0.034) in SKG mice compared to nontreated and isotype-treated animals (Figure 3B,C).

Furthermore, Safranin O/Fast Green-stained histological sections illustrate the differences in the extent of cartilage damage across the treatment groups (Figure 4A). In untreated and isotype control-treated mice, significant cartilage loss is evident, both in the metatarsus region (top panel) and in the lumbar intervertebral areas (bottom panel). These mice display severe cartilage degradation and joint morphology abnormalities. Conversely, mice treated with anti-VEGF-A antibodies exhibit markedly less cartilage destruction, with the ankle joint showing morphology closer to that observed in healthy controls. In the untreated group, pronounced inflammatory cell infiltration is visible in the metatarsus/ankle region, a feature notably absent in anti-VEGF-A treated mice. Furthermore, pronounced calcification is observed in the lumbar intervertebral spaces of untreated mice, with this pathological process also present but to a lesser extent in isotype-treated mice. In contrast, anti-VEGF-A-treated animals exhibit a lumbar spine morphology that closely resembles that of healthy controls. Qualitative assessment based on the OARSI cartilage damage score revealed that cartilage damage in the metatarsus region was significantly more severe in untreated and isotype-treated mice compared to anti-VEGF-A-treated mice (*p*=0.046 and *p*=0.035, respectively) (Figure 4B). Cartilage damage in the lumbar intervertebral regions was also lower in the anti-VEGF-A group compared to other mice groups with induced SpA-like disease. However, no statistically significant differences were observed between anti-VEGF-A and isotype-treated mice, while a significant difference was present between anti-VEGF-A and untreated groups (*p*=0.043) (Figure 4C).

The above results suggest that anti-VEGF-A treatment offers partial protection against cartilage damage but does not completely prevent inflammation or cartilage degeneration. This may be due to the timing of treatment initiation during disease progression rather than as a preventive measure. Moreover, these findings may reinforce the notion that SpA-like disease induced in SKG mice predominantly affects the peripheral skeleton, with less pronounced degenerative changes in the axial skeleton.

## 4. Discussion

This study demonstrates for the first time that anti-VEGF-A mAb therapy significantly reduces synovial inflammation, bone erosion, and pathological osteogenesis in the SKG mouse model of SpA. An important role of VEGF-A in the development and progression of inflammatory rheumatic diseases, including SpA, is recognized but not yet thoroughly established and studied. VEGF-A, as a pro-angiogenic cytokine, promotes angiogenesis by stimulating the proliferation, migration, and differentiation of endothelial cells and is expressed by a variety of cell types, including synovial fibroblasts, macrophages, and T cells [5, 22–24]. Several studies have shown that the concentration of VEGF-A is elevated in the synovial fluid and serum of patients with SpA compared to controls, particularly in those with active disease [25–28]. Given its central role in inflammation and angiogenesis, VEGF-A has been proposed as a potential therapeutic target in SpA [29].

Utilizing the SKG mouse model—characterized by peripheral arthritis, enthesitis, and spondylitis resembling human SpA [30]—we have demonstrated that anti-VEGF-A therapy targets core pathological features of the disease. This preclinical evidence underscores the therapeutic potential of anti-VEGF-A mAbs to modulate key pathogenetic mechanisms in SpA. In line with our findings, the use of anti-VEGF-A therapeutic antibodies has already shown promising results in several other animal models of rheumatic disorders, including osteoarthritis and collagen-induced arthritis [31–34]. Additionally, we have demonstrated the utility of the SKG model as a valuable tool to study particular aspects of SpA pathomechanisms, including the impact of potential novel therapeutic approaches on disease development and progression.

Although our study did not directly investigate the mechanisms underlying the effects of anti-VEGF-A therapy, the observed outcomes may be explained by previously proposed pathways, including (a) inhibition of neovascularisation, (b) reduction of inflammatory mediator expression and inflammatory cell migration, and (c) modulation of angiogenesis–immune crosstalk. The antibody-mediated inhibition of VEGF-A could be beneficial in slowing SpA progression, potentially through attenuation of new blood vessel formation in affected joints [35–38], thereby limiting the influx of inflammatory cells such as macrophages and T cells—the central players in SpA pathogenesis [39, 40]. Our recent study demonstrated that the expression of VEGF-A in classical monocytes is associated with the number of swollen and painful peripheral joints of SpA patients [41], suggesting that monocyte subpopulation-specific VEGF-A expression may substantially contribute to the development of peripheral arthritis (independently of the canonical prediction for inflammatory and anti-inflammatory cytokine production by nonclassical and classical monocytes, respectively [42]). Alongside the above, anti-VEGF-A treatment may stabilize vascular permeability, preventing excessive leakage from blood vessels, which could otherwise promote tissue edema and further inflammatory signaling. VEGF-A has been shown to increase vascular permeability facilitating additional recruitment of immune cells, which contributes to both inflammation and tissue damage [43]. This concept is further supported by findings from a proteoglycan-induced arthritis model, where intra-articular administration of the VEGF decoy receptor sFlt-1 led to reduced arthritis severity and macrophage infiltration, likely via VEGFR2-mediated suppression of vascular permeability and monocyte recruitment [44]. Furthermore, VEGF-A can influence the immune response by promoting the endothelial and immune cell-mediated release of pro-inflammatory cytokines such as IL-6 and TNF-*α*, both of which are central mediators in SpA pathogenesis [45, 46]. In support of this, IL-17A blockade in peripheral SpA patients has been shown to significantly reduce VEGF-A levels alongside other inflammatory and tissue remodeling biomarkers and to decrease the number of synovial high endothelial venules, underscoring the interplay between inflammation, angiogenesis, and structural damage [47]. Additionally, anti-VEGF-A therapy may act on bone remodeling, as the crosstalk between angiogenesis and the immune system is pivotal in driving the adverse bone damage and formation seen in these diseases [29, 48–51]. Anti-VEGF-A mAbs could disrupt this crosstalk, potentially slowing down or inhibiting bone tissue changes associated with SpA.

While our study demonstrates a reduction in inflammation following anti-VEGF-A treatment, it does not show complete abrogation of inflammation. This suggests that anti-VEGF-A therapy alone is not sufficient to control the complex inflammatory process in SpA. For instance, in an allograft study, anti-VEGF treatment markedly inhibited T-cell infiltration into allografts but, at the same time, failed to inhibit T-cell activation [52]. Thus, VEGF-A inhibition is likely to target the angiogenic aspect of inflammation, reducing vascular proliferation and immune cell infiltration into the joints, but it may not address other pathways driving inflammation, such as the activation of TNF-*α*, IL-17, or IL-23 signaling that are crucial for SpA pathogenesis. The possibility of combining VEGF inhibition with other targeted therapies, such as TNF-*α* inhibitors or IL-17 blockers, might provide a more comprehensive therapeutic approach. For example, dual inhibition of VEGF and TNF-*α* pathways has shown synergistic effects in reducing inflammation and tissue damage in experimental models of arthritis [53]. Additionally, IL-17 inhibitors, which directly target the Th17 T cells driving inflammation in SpA, could be combined with VEGF blockade to improve clinical outcomes [54]. In conclusion, anti-VEGF-A mAb therapy seems a promising option for the syndromic treatment of SpA by targeting vascular and inflammatory components of the disease, thus modulating the underlying pathological processes. The SKG model of SpA seems to be a valuable tool to prove therapy efficacy while having insight into SpA pathogenesis. The evidence from the studies utilizing animal models and human subjects highlights the growing interest in the anti-VEGF-A therapeutic approach. However, further research is needed to determine the optimal patient profiles, dosing regimens, and long-term safety of this potential therapy. Moreover, safety and long-term tolerability of anti-VEGF-A therapy in SpA patients remain an important consideration, as the use of anti-VEGF-A mAbs may cause systemic adverse effects, including hypertension, proteinuria, and risk of thromboembolic events seen in cancer patients [55, 56]. Moreover, treatment of cancer patients with anti-VEGF antibody bevacizumab has been associated with increased incidence of arthralgias. Studies have shown that up to 50% of patients receiving bevacizumab experienced joint pain, indicating a significant impact on their quality of life [57, 58]. This increase in joint pain raises important questions regarding the practical utility of VEGF-A inhibition in therapeutic settings, especially if the treatment does not adequately alleviate inflammation. The presence of arthralgias could indeed complicate treatment decisions and patient management, potentially leading to dose adjustments or even to discontinuation of the therapy. Nonetheless, as the understanding of the complex interplay between angiogenesis and inflammation in SpA continues to evolve, anti-VEGF-A treatment may provide important insight into the pathogenesis of SpA and become a valuable future addition to the current therapeutic options.

Nevertheless, this study has several limitations. First, although the SKG mouse model recapitulates key clinical and pathological features of human SpA, findings from animal models may not fully translate to human disease. Second, the treatment was initiated at disease onset, which may not reflect the therapeutic potential of anti-VEGF-A antibodies in established or chronic SpA. Third, the study did not include longitudinal functional or behavioral assessments, which could provide further insight into joint function and pain. Finally, potential systemic effects or off-target consequences of VEGF-A inhibition, including those affecting vascular integrity or immune regulation, were not assessed in this model. These limitations should be addressed in future studies to better understand the translational relevance and safety of VEGF-A-targeted therapies in SpA.

## Data Availability

All the data will be made available by the corresponding author upon reasonable request.
